# Narcotic Necessaries of Life

**Published:** 1848-09

**Authors:** 


*Narcotic Necessaries of Life.*—We quote the following from the *Dublin Medical Press*:—Editor Buff. Jour.

The following extract verifies our repeated predictions as to the ultimate effects to be expected from unrestricted indulgence in stimulants and narcotics:—

"Opium-Eating in England.—From the accounts of the Board of Trade, of the imports and exports of the united kingdom for the month ending May 5th, it would appear that the consumption of opium in this country is greatly on the increase. The quantity imported during the month amounted to no less than 7,029 pounds to 3,083 pounds imported during the same period in 1847. The total quantity of opium imported in 1847 amounted to 24,929 pounds. It is reported that the inmates of our workhouses are given to the practice of opium-eating; and the spreading of the so-called temperance principles may have tended to increase the demand of the drug."

Twenty-five thousand pounds of opium consumed in one year, in addition to a very liberal supply of alcohol in various forms, tobacco, hops, and other soothing appliances, is no small allowance! We speak of opium-eating now as a reproach; but was not tobacco-smoking a reproach, amongst Irish gentlemen, at least, some five-and-twenty or thirty years ago? It is not for us to attempt to correct this propensity to the enjoyment of narcotics in the world at large; we conclude that those who resort to them consider the relief of mental uneasiness by such means as justifiable as the relief of bodily pain by a similar method; all we consider ourselves called upon to allude to in connexion with the subject is the indulgence in the practice by medical students, amongst whom, as indeed amongst others at the same time of life, it is liable to degenerate into a dangerous vice. We express not merely our own opinions, but those of every man of right feeling engaged in the instruction of candidates for a place in our profession, that indulgence in the use of tobacco has had the most injurious effect in the medical schools. We do not mean to say that it leads universally, or perhaps generally, to habits of intemperance, or that it is the primary cause of delirium tremens in all cases, but we venture to assert that it is the fertile source of those evils. Tobacco-smoking leads to tippling, tippling to drunkenness, drunkenness to prostration of the intellectual faculties and every species of mental infirmity and derangement. Hence, as we believe, the deterioration of our medical schools, and the decline of that spirit of emulation which formerly pervaded them. We will not venture to follow up this tracing of disease to its remote cause, because people may think we are only indulging in a little concealed irony, but satisfy ourselves by appending the following extract by way of illustration:—

"Hypochondria Politica in Germany.—Dr. Brück, of Osnaburg, has just published, in Casper's *Wochenschrift*, a very interesting paper under the above title. The doctor gives a vivid description of the morbid effects of the recent violent political commotions in Prussia on a considerable portion of the population, which he contends had been previously the subjects of a kind of epidemic anœmia. In such constitutions late events had engendered a hypochondria nervosa, the result of the reflex action of violent emotions on different parts of the economy. The principal symptoms were, great paleness, anxious countenance, restlessness, and the skin inclined to constant moisture; cold extremities, diarrhœa, thirst, and white tongue. Appetite bad, except when excited by a stimulating diet; propensity for ardent spirits; copious discharge of pale urine in women, stoppage of the menstrual flux, increase of fluor albus, want of sleep, oppressive dreams, and general emaciation. Dr. Brück adds a few reflections, among which we extract the following: 'Twenty-one years ago, I mentioned, in a memoir on psychical medicine, that the numerous cases of hypochondria in our country among the higher and middle classes, were mainly owing to the want of interest in public affairs, and the total absence of coöperation between the citizens and the state. It is now evident that I was right, for the peculiar effects which the late political changes have produced, and from the nervousness with which we watch the events, prove that we have become more or less hypochondriacal. We are afraid of public life; all news of a political nature, which, amongst more advanced nations, are quietly discussed, has with us an immediate effect on the ganglionic system, frightens and unnerves us; the appetite disappears, sleep is disturbed, and the train of symptoms above mentioned make their appearance.'"

